# Potential and pitfalls of whole transcriptome-based immunogenetic marker identification in acute lymphoblastic leukemia; a EuroMRD and EuroClonality-NGS Working Group study

**DOI:** 10.1038/s41375-021-01154-z

**Published:** 2021-02-19

**Authors:** Vincent H. J. van der Velden, Monika Brüggemann, Giovanni Cazzaniga, Blanca Scheijen, Bastiaan Tops, Jan Trka, Karol Pal, Sonja Hänzelmann, Grazia Fazio, Simona Songia, Anton W. Langerak, Nikos Darzentas, EuroMRD, Vincent H. J. van der Velden, EuroClonality-NGS Working Group

**Affiliations:** 1 Department of Immunology, Laboratory Medical Immunology, Erasmus MC, University Medical Center Rotterdam, Rotterdam, The Netherlands; 2 Department of Hematology, University of Schleswig-Holstein, Campus Kiel, Kiel, Germany; 3 Centro Ricerca Tettamanti, School of Medicine, University of Milano Bicocca, Monza, Italy; 4 Department of Pathology, Radboud University Medical Center, Radboud Institute for Molecular Life Sciences, Nijmegen, The Netherlands; 5 Princess Máxima Center for Pediatric Oncology, Utrecht, The Netherlands; 6 Department of Pediatric Hematology and Oncology, University Hospital Motol, Charles University, Prague, Czech Republic; 7 Molecular Medicine Program, Central European Institute of Technology, Masaryk University, 62500 Brno, Czech Republic

**Keywords:** Acute lymphocytic leukaemia, VDJ recombination

## To the Editor:

Identification of immunoglobulin (IG) and T-cell receptor (TR) gene rearrangements in acute lymphoblastic leukemia (ALL) patients at initial presentation is crucial for monitoring of minimal residual disease (MRD) during subsequent follow-up and thereby for appropriate risk-group stratification. In a diagnostic setting, IG/TR gene rearrangements are generally identified using DNA-based PCR analysis, followed by classical Sanger sequencing or next generation sequencing (NGS) [1, 2]. Nowadays, whole transcriptome RNA sequencing (RNAseq) is frequently used to identify fusion genes and to assign patients into distinct molecular subgroups according to WHO 2016 classification, or for protocol-based clinical decisions [3]. Hence, it would be beneficial if RNAseq data could also be used for the identification of IG/TR gene rearrangements pertaining to the leukemic clone.

Recently, Yeoh and colleagues reported on the use of RNAseq data for identification of IG heavy chain (IGH) gene rearrangements, which was successful in approximately 90% of B-ALL patients [4]. Almost two-thirds of clonal IGH rearrangements were unproductive, whereas the vast majority (>98%) of background rearrangements were productive. Even though these data are promising, they are also incomplete since other IG/TR loci were not evaluated. Furthermore, these data underline that caution is warranted in the analysis of RNAseq data for IG/TR marker screening in ALL (and in other lymphoproliferative disorders requiring multiple RNA/DNA analyses): [5, 6] applying computational methods that only focus on productive rearrangements (e.g., like for most repertoire analyses) will clearly result in incomplete interpretation of IG/TR data for marker identification.

Within the EuroClonality-NGS Working Group and EuroMRD, we have developed, validated, and published assays for IG/TR DNA amplicon (‘DNAamp’ hereafter) and DNA capture-based analysis of relevant samples [1, 7, 8]. In line with these recent efforts, we decided to explore the possibilities and limitations of extracting data on IG/TR gene rearrangements from RNAseq data. Here we report on a complete but preliminary analysis of all IG/TR loci from RNAseq data, using DNAamp data as benchmark.

RNAseq data from 165 ALL patients at time of diagnosis were obtained using Illumina TruSeq Library Prep or Universal RNAseq kit (Nugen, Tecan) and sequenced by Illumina Next-Seq or Novaseq (2 × 75 bp). DNAamp IG/TR data were obtained from the same patients using the aforementioned assays developed by EuroClonality-NGS, which employ separate primer sets: IGHV-IGHD-IGHJ, IGHD-IGHJ, IGK (often split in two tubes: IGKV-IGKJ/Kde and intronRSS-Kde), TRBV-TRBD-TRBJ, TRBD-TRBJ, TRG, TRD—note the absence of an IGL and TRA primer set [1, 7]. The ARResT/Interrogate bioinformatics pipeline [9], which has also been developed and validated within EuroClonality-NGS [7, 9, 10], was used to produce profiles of 22 gene IG/TR rearrangement types, or junction classes, both complete and incomplete, (potentially) productive and unproductive, across all seven IG/TR loci: IGH, IGK, IGL, TRA, TRB, TRG, and TRD (see Table 1). Rearrangements were organized (e.g., for calculating their abundance) by junction class (rearrangement type), 5′ gene, junctional segmentation and N-(D)-N region statistics (5′ gene deletions, N-(D)-N length, 3′ gene deletions), 3′ gene, and junction amino acid sequence [7]. We only allowed IG/TR rearrangements appearing uniquely across cases to minimize potential contamination and artefacts. For identifying potential MRD markers in DNAamp data, we required a minimum abundance of 10 reads and 5% of reads (amplified in separate tubes by the corresponding EuroClonality-NGS primer set) [1].

**Table 1:** IG/TR rearrangements detected by DNA amplicon-based methods (DNAamp) and by RNAseq.

	Rearrangements/case^a^			RNAseq MRD markers according to DNAamp benchmark^b^		
	DNAamp	RNAseq (“damaged”)	RNAseq Productive	DNAamp	RNAseq	RNAseq (%)
IGH complete VJ:Vh-(Dh)-Jh	2241	643 (215)	364	223	188	84.3
IGH incomplete DJ:Dh-Jh	1407	21 (9)	na	89	69	77.5
IGK complete VJ:Vk-Jk	762	436 (61)	323	63	23	36.5
IGK other Vk-Kde	285	<1	na	57	2	3.5
IGK other intron-Kde	780	<1	na	45	1	2.2
IGL complete VJ:Vl-Jl	na^c^	329 (53)	233	na		
TRA complete VJ:Va-Ja	11	62 (20)	25	0		
TRA + D complete VJ:Va-Jd	10	<1	<1	0		
TRA + D complete VJ:Vd-(Dd)-Ja	133	2 (1)	<1	33	14	42.4
TRA + D incomplete DJ:Dd-Ja	71	37 (25)	na	27	1	3.7
TRA + D incomplete VD:Va-Dd3	na	<1	na	na		
TRB complete VJ:Vb-(Db)-Jb	1921	128 (34)	72	101	53	52.5
TRB incomplete DJ:Db-Jb	1555	13 (6)	na	71	19	26.8
TRB incomplete VD:Vb-Db	na	1 (<1)	na	na		
TRB other DD:Db-Db	na	<1	na	na		
TRD complete VJ:Vd-(Dd)-Jd	184	2 (<1)	1	17	4	23.5
TRD incomplete DJ:Dd2-Jd	139	1 (1)	na	11	1	9.1
TRD incomplete DJ:Dd3-Jd	na	<1	na	na		
TRD incomplete VD:Vd-Dd3	188	<1	na	82	1	1.2
TRD other DD:Dd2-Dd3	74	0	na	36	0	0
TRD other DD:Dd3-Dd2	na	<1	na	na		
TRG complete VJ:Vg-Jg	2163	12 (2)	3	198	91	46

^a^Average number of rearrangements per case as detected by DNAamp and RNAseq. The RNAseq data refer to total number of rearrangements (with in between brackets the number of “damaged” sequences) and to the number of productive rearrangements. A total of 165 ALL patients were evaluated.

^b^Number of potential MRD markers identified by DNAamp (as per abundance thresholds), and number and percentage of those markers identified by RNAseq at any abundance. A total of 165 ALL patients were evaluated.

^c^na: not analyzed/not applicable.

**Fig. 1:**
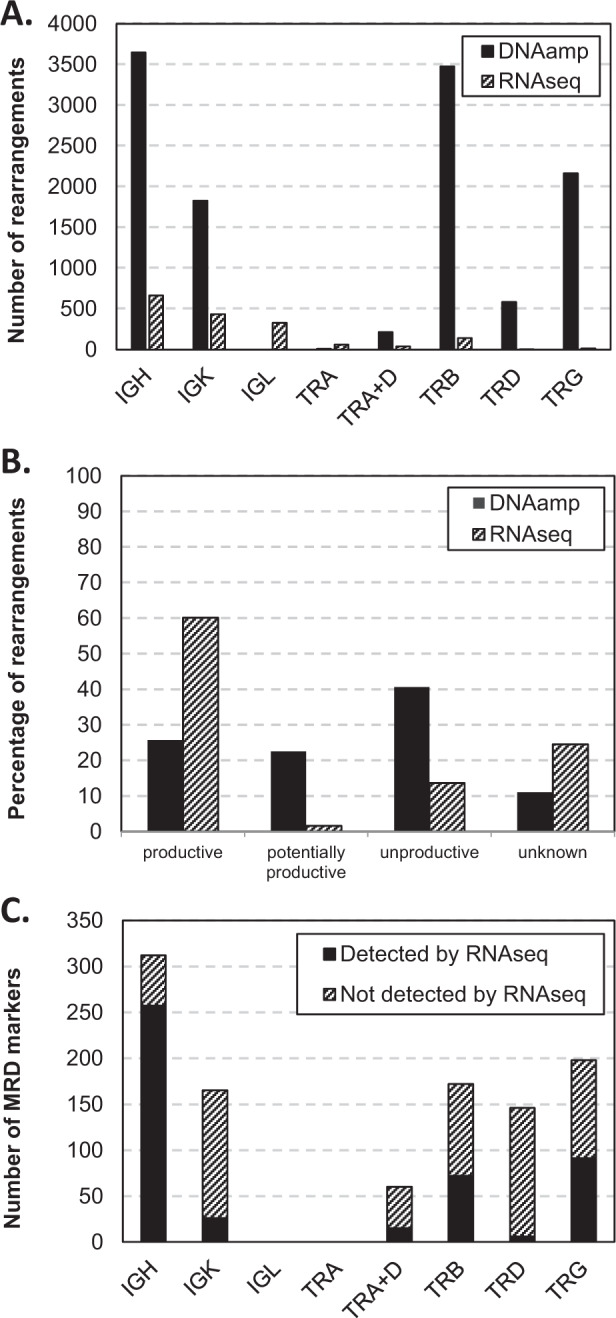
Comparison between IG/TR rearrangements detected by amplicon-based assays (‘DNAamp’) and RNAseq in 165 ALL patients. **A** Average number of rearrangements detected for the various IG/TR loci per case. **B** Frequencies of productive, potentially productive (incomplete sequences that have the potential to be productive when they would be complete), and unproductive rearrangements. Unknown: non-VJ and non-DJ classes, and “damaged” rearrangements from RNAseq. **C** Number of RNAseq re-identifiable MRD markers per target according to DNAamp benchmark.

Comparison of DNAamp and RNAseq IG/TR data revealed clear differences in the number of identified IG/TR rearrangements (summarized in Fig. 1 and Table 1). Importantly, using an RNAseq read length of ~150 (2 × 75bp), around 25% of RNAseq rearrangements are missing part of the junctional residues (herein “damaged”) when the fragment is longer than the read coverage. Apart from the expected absence of TRA and IGL events from the DNAamp data, we observed additional differences in the average absolute number of rearrangements per case for each locus (Fig. 1A): the DNAamp data provide an average of 1703 rearrangements per case per locus (range 11–3648) compared to 211 (range 3–664) for RNAseq. When “damaged” rearrangements were ignored, RNAseq numbers fell to 157 (range 2–440). The most noticeable difference in relative incidence is the remarkable under-representation of TRB, TRD and TRG gene rearrangements in the RNAseq data. Furthermore, of the rearrangements identified by RNAseq, the vast majority is complete (96%) and the absolute majority is productive (>60%; Fig. 1B). In contrast, by DNAamp the complete rearrangements are relatively less frequent (~60%) and nonproductive rearrangements are found in about 40% of patients.

The lower number of total IG/TR rearrangements detected by RNAseq as compared to DNAamp also limited the identification of potential MRD markers. Figure 1C and Table 1 show the absolute number of potential MRD markers above the aforementioned abundance thresholds in the DNAamp data, and results from using DNAamp as benchmark for the sensitivity of RNAseq to identify MRD markers. At present, the non-trivial question of appropriate thresholds for marker screening in RNAseq has not been answered. Therefore, we decided to search for the respective MRD marker obtained by the DNAamp approach in the RNAseq data at any abundance. This probably means that our results are overestimating the ability of RNAseq to recover DNAamp markers. Overall, less than half of the DNAamp markers were found in the RNAseq data (467/1053; 44%). Best concordance was observed for IGH (complete and incomplete) markers (82% of DNAamp markers also identified by RNAseq); other complete markers (IGK, TRB, TRA + D, TRD and TRG) were partially detected by RNAseq (24–52%), while most incomplete markers and virtually all IGK-Kde markers were only limitedly identified by RNAseq (<10%). Evaluation of the individual ALL cases showed that in eight out of 165 cases (5%) none of the DNAamp markers were found in the RNAseq results (again, at any abundance), while identical marker profiles were seen in only 19/165 cases (12%). In the remaining 138 patients, at least some of the potential markers were missed. When the same abundance criteria were used for both DNAamp and RNAseq, by RNAseq, 38 cases (23%) had one potential marker, 50 cases (30%) two, and 37 cases (22%) three (and seven cases had none); for DNAamp these numbers were six (4%), three (2%), and 14 (8%), respectively. The remaining cases (i.e., 33 (20%) in RNAseq, 142 (86%) in DNAamp) had four or more potential markers available. Thus, at least 2 IG/TR markers (required in most current clinical protocols) were identified by DNAamp in 159/165 (96%) cases and by RNAseq in 120/165 cases (73%).

Our preliminary data thus show that IG/TR rearrangements detected by DNA amplicon-based methods are clearly distinct from RNAseq-identified rearrangements. Obviously, this is to a large extent explained by the underlying (immuno)biology of IG/TR gene rearrangements, which will mainly be transcribed if complete and productive, thus allowing production of a functional IG/TR chain. In contrast, non-transcribed unproductive and incomplete rearrangements are hardly or not detectable by RNAseq. Of importance, about 70% of IGH rearrangements in ALL patients are unproductive, whereas non-leukemic rearrangements generally are productive [4, 6]. While it is appropriate to filter out out-of-frame immune rearrangements in analysis of functional repertoires, this is certainly not appropriate for ALL IG/TR marker analysis [5]. In our analysis, we did not yet take full transcription levels into account (i.e., we counted in de-duplicated reads, or unique fragments, as in DNA capture analyses), but it will be interesting to evaluate whether for example TR rearrangements in reactive T-cell clones show differences in transcript levels compared to cross-lineage TR rearrangements in B-cell precursor type ALL. From a technical perspective, it should be noted that we applied 2 × 75 bp sequencing and it may be expected that IG/TR rearrangement detection will be improved if longer reads are used. From a clinical perspective, RNAseq data may be used for MRD marker identification in those cases where DNAamp methods are unsuccessful or unavailable, if appropriate bioinformatic strategies are used.

Of note, IG/TR rearrangements may also be derived from whole-exome sequencing (WES) or whole-genome sequencing (WGS) datasets that, in contrast to RNAseq data, do not depend on the transcriptional level of rearrangements. This creates a clear advantage as was recently showcased in work introducing IgCaller for WGS-derived IGH data [11].

We therefore plan to continue and expand on the evaluation of not only RNAseq, but also WES and WGS data, mainly in ALL but also in NHL, in comparison to results obtained from a large set of DNAamp and targeted DNA capture data from the EuroClonality-NGS/EuroMRD groups. Challenges will include compiling appropriate rules and thresholds for marker identification, preparing specific protocols and suggesting limits for the safe use for each technology, for example and especially for MRD monitoring.
